# Computational identification of microbial phosphorylation sites by the enhanced characteristics of sequence information

**DOI:** 10.1038/s41598-019-44548-x

**Published:** 2019-06-04

**Authors:** Md. Mehedi Hasan, Md. Mamunur Rashid, Mst. Shamima Khatun, Hiroyuki Kurata

**Affiliations:** 1 Department of Bioscience and Bioinformatics, Kyushu Institute of Technology, 680-4 Kawazu, Iizuka, Fukuoka, 820-8502 Japan; 2 Biomedical Informatics R&D Center, Kyushu Institute of Technology, 680-4 Kawazu, Iizuka, Fukuoka, 820-8502 Japan

**Keywords:** Protein analysis, Protein function predictions

## Abstract

Protein phosphorylation on serine (S) and threonine (T) has emerged as a key device in the control of many biological processes. Recently phosphorylation in microbial organisms has attracted much attention for its critical roles in various cellular processes such as cell growth and cell division. Here a novel machine learning predictor, MPSite (Microbial Phosphorylation Site predictor), was developed to identify microbial phosphorylation sites using the enhanced characteristics of sequence features. The final feature vectors optimized via a Wilcoxon rank sum test. A random forest classifier was then trained using the optimum features to build the predictor. Benchmarking investigation using the 5-fold cross-validation and independent datasets test showed that the MPSite is able to achieve robust performance on the S- and T-phosphorylation site prediction. It also outperformed other existing methods on the comprehensive independent datasets. We anticipate that the MPSite is a powerful tool for proteome-wide prediction of microbial phosphorylation sites and facilitates hypothesis-driven functional interrogation of phosphorylation proteins. A web application with the curated datasets is freely available at http://kurata14.bio.kyutech.ac.jp/MPSite/.

## Introduction

Protein phosphorylation is one type of post-translational modification (PTM) that plays an important role in regulating many signal transduction pathways^1–4^. Since 1932^5,6^ eukaryotes have been extensively studied and most of the identified phosphorylation acceptor residues were serine (S), and threonine (T). Different studies suggest that phospho-serine (pS) and phospho-threonine (pT) residues are critical for functional regulation and signaling transduction^6–10^. Recently, some leading studies have expanded an understanding of molecular mechanisms and functional roles in microbial phosphorylation^6,11–15^. Although the experimental determination of microbial phosphorylation proteins with specific modified sites is increasing in the present era, the mechanism of phosphorylation specificity is still largely unknown until now^7,12,16^. Therefore, identification of microbial phosphorylation sites is necessary for further elucidating the mechanism of phosphorylation.

Due to the potential significance of microbial phosphorylation, identifying the microbial phosphorylation sites in proteins is a prerequisite and offers valuable evidence in biomedical research. The experimental identification of the phosphorylation sites is necessary and mainly depends on mass spectrometry analysis which is laborious and expensive. Before the experimental investigation, computational modeling of microbial phosphorylation sites based on protein sequence information is therefore highly desired.

Although there are a large number of computational studies in higher organisms^17–19^, predictions in microbial cells are still rare. To date, two computational methods were proposed to predict microbial phosphorylation sites^20–22^. Initially, Miller *et al*. developed NetPhosBac, which was constructed by an artificial neural network^21^. Li *et al*. proposed a predictor cPhosBac based on the composition of the *k*-spaced amino acid pair (KSAAP) combined with motif length selection^20^. Until now, the NetPhosBac, which is publicly available, predicted pS and pT residues of microbial phosphorylation sites. However, the overall performance of the aforementioned existing predictors is still not satisfying and there is further room to improve the prediction performance.

In this article, a new computational method of the MPSite was developed, which predicts pS and pT residues of microbial phosphorylation from the protein sequences. We investigated multiple sequence features including amino acid properties, evolutionary, and structural features to represent the peptide fragments of phosphorylation sites. We optimized the feature models via a Wilcoxon rank-sum test (WR). Then the final feature vectors were classified by a random forest (RF) classifier. To assess the robustness and prediction accuracy of the MPSite, 5-fold cross-validations (CV) and independent tests were adopted. The MPSite outperformed other existing prediction models, suggesting that the MPSite is a useful computational resource to identify pS and pT sites in microbes.

## Materials and Methods

### Data preparation

We collected microbial PTMs of pS and pT sites from the dbPSP database^6^. The pS and pT containing peptides experimentally detected without any phospho-groups were used as negative samples. Each site was represented as a peptide segment of 21 (±10) residues with S and T in the center. Homology reduction was performed on the full protein length using CD-HIT with a default value of 30% sequence identity threshold^23^. At first, after removing redundant sequences, we assessed the performance of a pS site prediction classifier. Since the performance of the predictive model might be overestimated by an overfitting of the training dataset, an independent test data set, definitely blind to the training set, was collected. The dataset for independent test was collected by random selection from the final dataset (2,045 positive sequences and 34,519 negative sequences). We adjusted the ratio of negative examples to positive ones to 2:1, because the performance of machine learning methods is often deteriorated by unbalanced datasets that differ in orders of magnitude^24^. Consequently, the training dataset contained 1,704 positive and 3,408 negative sequence fragments; the independent test dataset contained 341 positive and 682 negative data. The above dataset selection procedure was applied for protein acetylation and S-sulfenylation site prediction in the previous literatures^25,26^.

Similarly, we assessed the performance of a pT site prediction classifier. After 30% sequence redundancy removal, the training dataset consisting of 1,401 positive and 2,802 negative sequence fragments were extracted from the final dataset (1,655 positive sequences and 24,963 negative sequences). The final independent test dataset contained 254 positive and 508 negative samples. All of these curated datasets are available at http://kurata14.bio.kyutech.ac.jp/MPSite/.

### Overall workflow

**Figure 1:**
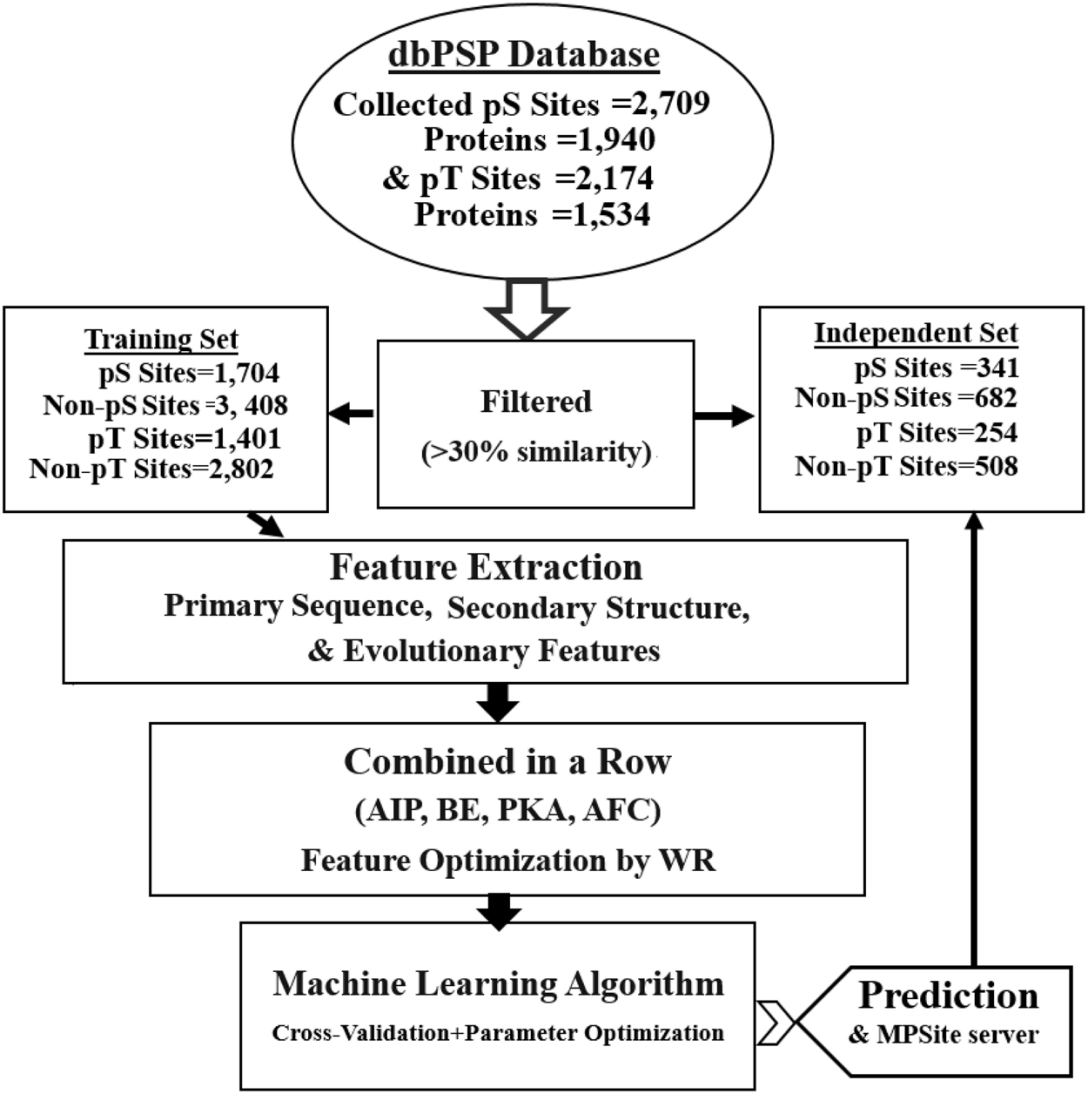
A computational framework of MPSite.

An overall framework of the MPSite predictor is shown in Fig. 1. Firstly, a sequence window of ±10 amino acids that possesses a positive/negative samples with S/T in the center was encoded in four different approaches. The optimum encoded feature vectors were combined in a row into one feature vector. Ultimately, the final feature vectors were optimized by the WR method via an RF classifier. Then, a confident cutoff was considered to identify the pS and pT sites.

### Sequence encoding strategies

To establish an accurate machine learning (ML)-based prediction model, the individual sequence fragment was encoded into a numeric feature vector. It is a critical step to represent the collective classifiers. Therefore, to obtain the local information around the microbial phosphorylation site, a high-quality sequence encoding method was essential. As a substitute for retaining a general binary representation of corresponding amino acid sequences, different types of encoding methods were investigated, including amino acid composition (AAC), amino acid frequency composition (AFC), binary encoding (BE), amino acid index properties (AIP), secondary structural feature (SSF), position-specific scoring matrix (PSSM), and profile based *k*-space amino acid pair composition (PKA), as follows.

(i) AAC encoding
  The AAC encoding is widely used for protein bioinformatics research^27,28^. When a fragment sequence is composed of 20 amino acids, it contains 20-dimensional features.
(ii) AFC encoding
  By effectively representing the short sequence motif information in protein sequences or fragments, AFC is an important encoding scheme in many prediction tasks^27–29^. In this method, possible optimum pairs are collected from the fragment sequences. A 2,205-dimensional feature vector was generated in the AFC encoding scheme. Details in the AFC encoding are described in our previous studies^30^.
(iii) BE encoding
  The binary encoding scheme was generated by a 20-dimensional binary vector for each residue in a sliding window^31^. A sequence fragment of 420-dimension (21 × 20 = 420) feature vector was obtained through binary encoding.
(iv) AIP encoding
  The AIP database (version 9.1) has the numerical indices of physicochemical and biochemical properties of amino acids^32^. After evaluating the different types of AIP, we selected 15 informative amino acid indices (Table S1), including BLAM930101, MAXF760101, TSAJ990101, NAKH920108, CEDJ970104, LIFS790101, NOZY710101, HUTJ700103, NAKH900109, BIOV880101, MIYS990104, PUNT030101, WOEC730101, BASU050102, and SUYM030101. These properties were transformed into the positive and negative samples for generating the feature vectors. The gap and pseudo amino acids were encoded as 0. In a sequence fragment, a 315-dimension (21 × 15 = 315) feature vector was obtained through AIP encoding.
(v) SSF encoding
  The SSF features are generated by the SPIDER2 software that is widely used in bioinformatics research^33,34^. Three types of SSF features are generated by SPIDER2: accessible surface area, backbone torsion angles (BTA) and secondary structure (SS). The BTA generates 4-type feature vectors of phi, psi, theta and tau. The SS generates 3-type feature vectors of helix, strand, and coil. Totally, 8-type feature vectors are generated. As a result, for each fragment sequence, 168 (21 × 8) dimensional feature vectors were generated.
(vi) PSSM encoding
  The PSSM profile was generated by using PSI-BLAST (version 2.2.26+) against the whole Swiss-Prot non-redundant database (December 2010) with two default parameters: e-value and iteration times of 1.0 × 10^−4^ and 3, respectively^35,36^. Then, we extracted the feature vectors using a sliding sequence window. The dimension of the PSSM profile for each sequence fragment was (21 × 20) = 420.
(vii) PKA encoding

After generating the PSSM profile, we generated possible *k*-space pair composition from the PSSM, i.e., PKA, in the same manner as the previous study of protein pupylation site prediction^30^. When an optimal *k*-space was 0, 1, 2, 3, and 4, a (5 × 20 × 20 = 2,000) dimensional feature vector was generated.

### Feature selection

Note that the proposed method contains high dimensional features. In the sequence of the given protein, the conservative possessions vary from site to site. As a result, near the central sites some residues a little contribute to the identification of PTM sites^37,38^. To characterize the relative importance and contribution of each initial feature, the WR algorithm, a well-established feature extraction method, was considered. It can rank all the initial features according to their relevance to the redundancy between the features themselves and the response variables. Details are described in elsewhere^39^.

### Combined model

To enhance the performance of the MPSite, we combined the optimum encoding features. In this study, AFC, AIP, BE, and PKA schemes performed better than others. Therefore, we combined these four schemes in a row. For instance, AFC, AIP, BE and PKA have 2,205, 315, 420, and 2,000 dimensional feature vectors, respectively. The combined feature vector was 4,940 dimensional.

### Machine learning algorithm

A supervised ML algorithm, RF, was employed^40^. The RF is one of the most precise ML algorithms and provides highly accurate classification results in bioinformatics research^31,41–43^. RF works as an ensemble and de-correlated decision trees, which ‘votes’ for one of the two classes, either positive or non-negative samples. The experimentally verified phosphorylation samples were labeled ‘+1’, while the other lysine residues labeled ‘−1’. Based on the positive and negative samples, four different types of features were generated using a series of input feature encodings. These generated features were input into the RF classifiers to identify whether they are positive or negative samples.

In this study, the performance of the RF was characterized in comparison to four commonly used ML algorithms: Naive Bayes (NB)^28^, decision trees (DT), SVM^30^, and artificial neural network (ANN)^28^. We used the NB, DT, and ANN algorithms of the WEKA software^44^ and the SVM algorithm with a kernel radial basis function of the LIBSVM package (http://www.csie.ntu.edu.tw/Bcjlin/libsvm/). To examine the optimal parameters, the grid search approach evaluated by a 5-fold CV test.

### Performance matrix

To evaluating the performance of the proposed method, four necessary yardstick statistical measurements were used: accuracy (*Ac*), sensitivity (*Sn*), specificity (*Sp*), Matthews’ correlation coefficient (*MCC*), and area under the ROC curves (AUC), as follows.

1 $$Ac=\frac{TP+TN}{TP+FN+FP+TN}$$

2 $$Sn=\frac{TP}{TP+FN}$$

3 $$Sp=\frac{TN}{TN+FP}$$

4 $$MCC=\frac{TP\times TN-FP\times FN}{\sqrt{(TN+FN)\times (TP+FP)\times (TN+FP)\times (TP+FN)}}$$

where *TP, TN, FP*, and *FN* denoted the numbers of true positives, true negatives, false positives and false negatives, respectively. The receiver operating characteristics (ROC) curve (*Sn* vs. (1 − *Sp*) plot) was drawn. Different thresholds were considered to plot the ROC curves. The AUC values were calculated by the pROC R-package^41,42^.

## Results and Discussion

### Analysis of amino acid preferences in microbial phosphorylation sites

**Figure 2:**
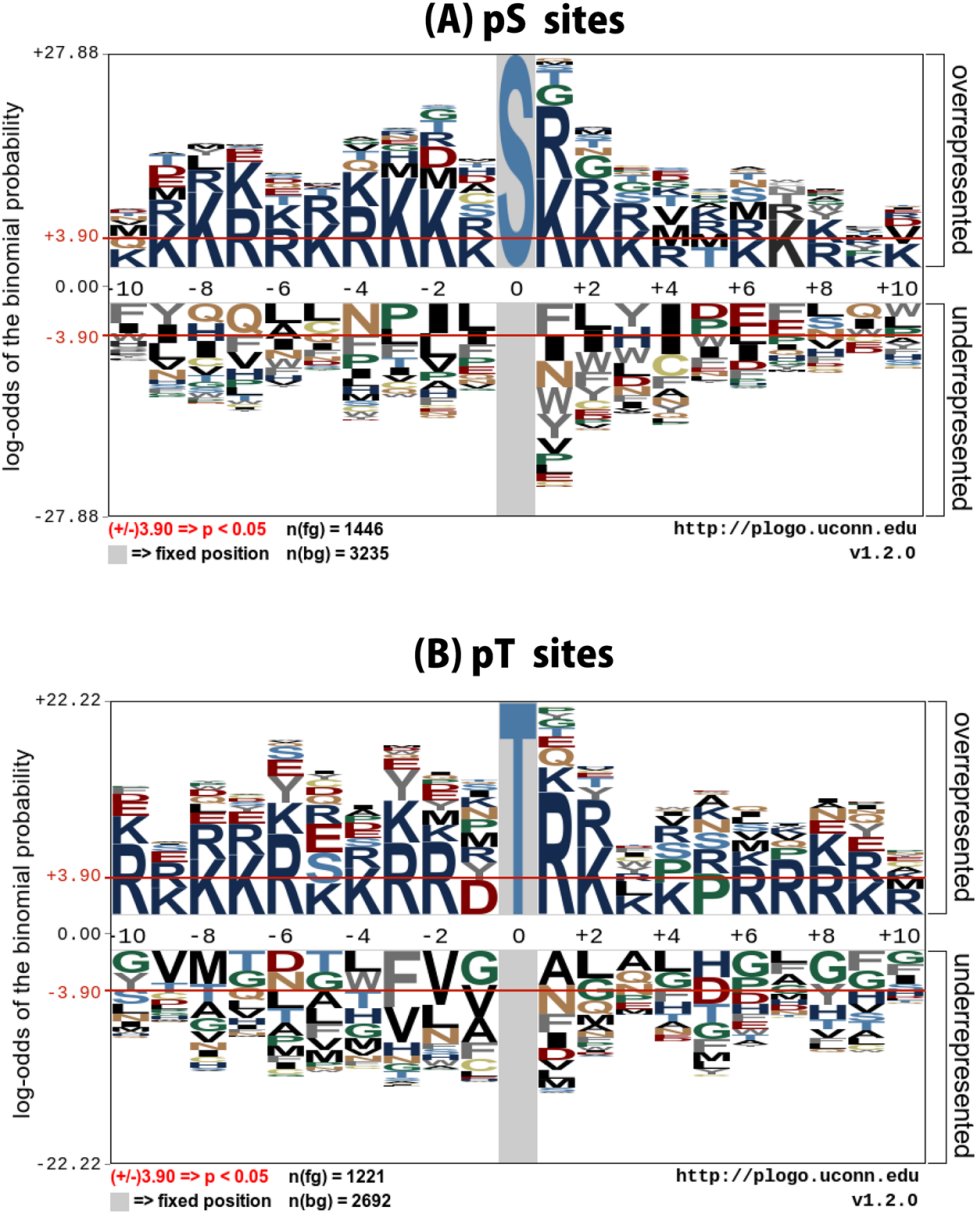
Sequence logo representation of pS and pT sites. The local sequence neighborhood of 10 upstream and 10 downstream residues surrounding the phosphorylation sites was used to plot the sequence logos. Two-sample logos show the dominance of surface accessible residues in microbial pS and pT sites.

To understand informative features surrounding pS and pT residues of microbial phosphorylation sites, we examined the flanking sequences of microbial phosphorylation with the pLogo program (https://plogo.uconn.edu/), a hypothetical approach to identifying the presence of sequence motifs. In the sequence logos, the residue heights were scaled according to their statistical significance, while the red bar signifies a statistical significance threshold of *p* = 0.05. In Fig. 2A, it can be seen that Lys (K) at positions −10, −9, −8, −5, −3, −2, −1, +1, +2, +3, +6, +7, +9, and +10, and Arg (R) at positions −7, −6, −4, +4 and +8 were significantly overrepresented compared with other amino acids, while Leu (L) at positions −6, −5, −1, and +2, Gln (Q) at positions −8, −7 and +9, Phe (F) at position −10, +1, and +7 and Tyr (Y) at −9 and +3 were significantly underrepresented.

In Fig. 2B, Lys (K) at positions −9, −8, −7, −5, −4, +2, +3, +4, and +9, and Arg (R) at positions −10, −6, −3, −2, +1, +6, +7, +8 and +10 were significantly overrepresented compared with other amino acids, while Ala (A) at positions +1 and +3, Gly(G) at positions −10, −1, +6, +8, and +10, Leu (L) at positions −4, +2, +4 and +7, Phe (F) at position −3 and +9 and Thr (T) at −7 and −5 were significantly underrepresented. Notably, it can be seen that a predominant characteristic of microbial S and T sites of phosphorylation is the requirement of R and K residues at the enriched position, which might be responsible for the creation of bends or flexibility in the pS and pT sites. Amino acid preference difference between two samples can explain how the feature vector combining method achieves a reasonable performance.

### Overall performances of the MPSite in training datasets

Firstly, we developed the models for discriminating pS and pT sites from the dbPSP dataset using profile-based methods including the PSSM and KPSSM methods. The RF-based models have been developed using a sparse profile of patterns, which is represented by a vector length of W × 20 (W is the sequence fragment length, 21 in this study). The model performances were measured using 5-fold CV test via the RF classifier. We achieved AUCs of 0.608 and 0.691 for PSSM and PKA, respectively in pS site prediction **(**Table 1**)**. In a similar way we examined pT site prediction achieved AUCs of 0.616 and 0.813 for PSSM and PKA, respectively.

**Table 1:** AUC value of different schemes on the training dataset via a 5-fold CV test.

Method	pS-site	pT-site
AAC	0.639	0.646
PSSM	0.608	0.616
PKA	0.691	0.813
AIP	0.671	0.685
BE	0.683	0.690
AFC	0.725	0.826
SSF	0.641	0.662
MPSite	0.822	0.853
DT	0.807	0.817
SVM	0.819	0.838
NB	0.789	0.811
ANN	0.774	0.784

The AUC scores of AAC, PSSM, PKA, AIP, BE, AFC, and SSF schemes were measured by using the RF algorithm. The AUC value of MPSite, DT, SVM, NB, and ANN are estimated by integrating the four descriptors of PKA, AIP, BE, and AFC.

Secondly, we developed an AIP property-based model. We tested 15 AIP properties (Table S1), which were shown to be a good index for pS and pT site prediction. The model achieved AUC values of pS and pT sites of 0.671 and 0.685, respectively **(**Table 1**)**. The AFC methods performed the best for all the single encodings, with AUCs of 0.725 and 0.826 for pS and pT site prediction, respectively (Table 1). We generated the SSF features using 8 types of properties (Material and Methods) and evaluated these features using 5-fold CV test through training datasets. The SSF model achieved AUCs of 0.641 and 0.662 for pS and pT sites, respectively **(**Table 1**)**. The above analyses demonstrated that the four methods of AIP, BE, AFC, and PKA were better than any other single encoding methods for predicting pS and pT sites.

**Figure 3:**
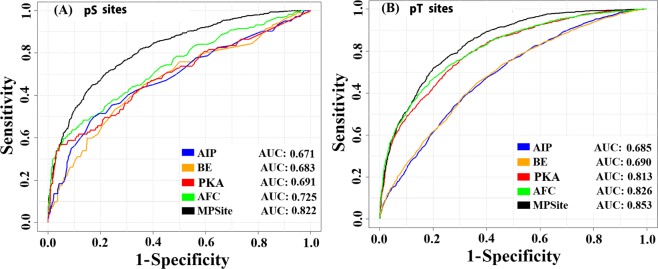
ROC curves on the various prediction models using a 5-fold CV test on training datasets. (**A**) Performance in the pS site dataset and (**B**) Performance in the pT site dataset. ‘MPSite’ indicates the optimum performances of the combined four features via the WR scheme.

Finally, we tested the MPSite that combined the four AFC, AIP, BE, and PKA encoding methods. These four feature vectors were directly combined in a row for prediction of pS sites. After combining these features, the total dimension was 4,961, renamed as “all-features”. Then, we optimized these features using the WR scheme. After several trials, the top 1,500 feature vectors were collected from the all-features for pS site prediction. The collected feature vectors were transformed into a new ordered feature based on low to high WR values. The final feature vectors were trained by the RF classifier. The optimum RF decision trees were grown up through the training dataset based on the 5-fold CV. The MPSite provided the highest AUC value of 0.822 (Fig. 3A). The performance indexes in terms of *Sp, Sn, Ac*, and *MCC* were 0.897, 0.503, 0.766, and 0.452, respectively, in the training dataset (Table 2). The performances of the combined model surpassed those of the single encoding methods (Fig. 3A).

**Table 2:** Performance of MPSite based on the training datasets via a 5-fold CV test.

Predictors	pS sites	pT sites
*Sp*	0.897	0.901
*Sn*	0.503	0.596
*Ac*	0.766	0.799
*MCC*	0.452	0.522
AUC	0.822	0.853

In the pT site prediction model, after combining the four encoding features, we collected top 2,100 feature vectors by the WR via the training datasets through 5-fold CV test. These optimum features were trained the RF classifier. The highest *Sp, Sn, Ac*, and *MCC* were 0.901, 0.596, 0.799, and 0.522, respectively (Table 2). Moreover, we depicted the ROC curve for each method and their combined model (Fig. 3B). The pT site prediction performance of the MPSite surpassed those of the single encoding methods, as well as the pS site prediction.

**Figure 4:**
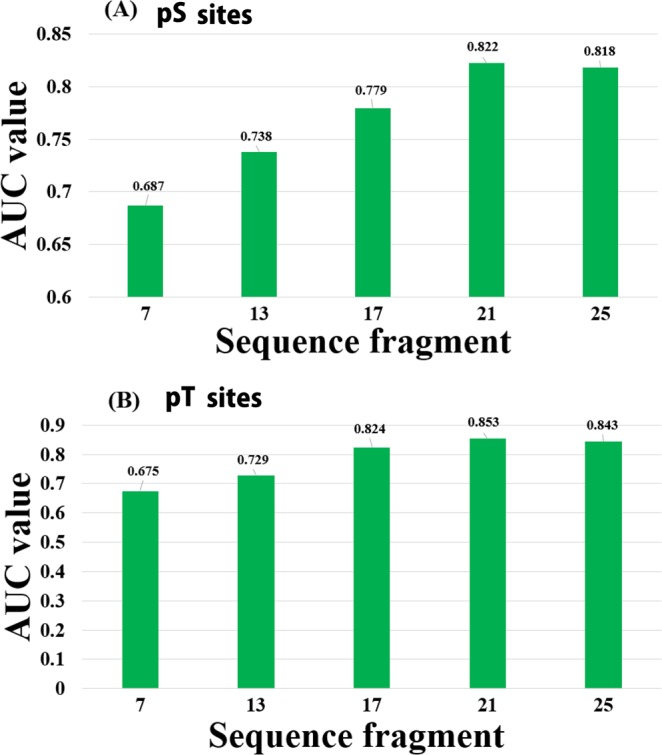
AUC values for different window sizes based on 5-fold cross-validation tests. (**A**) pS and (**B**) pT site prediction.

Moreover, in order to estimate the influence of the surrounding residues, the window fragments were optimized based on AUC values by using the training datasets. To assess the sequence similarity of the diverse region around the phosphorylation sites, we changed a windows size from 7 to 25 in both the pS and pT classifiers based on all features. An optimal window size of 21 was selected **(**Fig. 4**)**. In addition, we examined the effect of different positive versus negative samples in the training dataset on the prediction performance of the MPSite. The performance of the pS and pT models trained with different positive to negative sample ratios was estimated by 5-fold CV test, as shown in Table S2. A 1:2 ratio of the positive versus negative samples showed high *Sn*, *MCC* and AUC values compared with the other sample ratios for both the pS and pT classifiers. An increase in negative samples the *Ac* increased, but slightly decreased the *Sn*, *MCC* and AUC values probably due to the imbalanced datasets.

### Feature significance analysis

To investigate the significant features, we collected the top 30 features and their corresponding scores through the WR method from all-features for pS sites in Table S2. The selected features were found significant for the positive and negative samples (by the two-sample *t*-test *p* < 0.01). Moreover, we revealed that the four types of features of AFC, AIP, PKA, and BE were included in the top 30 significant features (Table S3). This analysis suggested that a combination of four types of features is critically responsible for pS site prediction.

Furthermore, we collected top 30 features ranked and their corresponding scores through the WR method for pT sites (Table S4). We found the significant difference between the positive and negative samples on the top 30 features (by the two-sample *t*-test p < 0.05). Interestingly, we found that the four types of features (AFC, AIP, PKA, and BE) were included in the top 30 significant features. In the above analysis, we concluded that the combination of features, AFC, AIP, PKA, and BE was particularly important for model performance in pS and pT site prediction.

### Performance of different ML algorithms on training datasets

The performance of the RF was compared to the four widely-used machine learning algorithms of DT, NB, SVM, and ANN by using the same training features as selected in the previous section for pS and pT site prediction. The AUC values of the prediction by the four algorithms, calculated by 5-fold CV test, are listed in Table 1. The RF algorithm provided higher AUC than any other algorithms, while the SVM performance was comparative to the RF (Table 1).

### Performance evaluation with existing algorithms using the independent datasets

The performance evaluation of different schemes is often difficult because they use different training samples with different ratios of positive to negative datasets and diverse assessment procedures. We evaluated the predictive performances of the NetPhosBac^21^ tool using the independent dataset, while the cPhosBac^20^ is not publicly available. The NetPhosBac implements pS and pT site prediction classifiers. Initially, we compared the MPSite with the NetPhosBac and the four ML-based predictors for pS sites. Each model was characterized in terms of *Sn, Sp, Ac*, and *MCC*. As shown in Table 3, the MPSite (*Sp* = 0.811, *Sn* = 0.412, *Ac* = 0.678, and *MCC* = 0.239) greatly outperformed the NetPhosBac and the four ML-based algorithms. Indeed, all performance measures in the MPSite were higher than those of the other methods, thus indicating the superiority of the MPSite in pS site prediction. Next, we compared the performances of the MPSite with those of the NetPhosBac and the four ML-based models using pT sites. The MPSite scheme presented the highest *Sn, Ac*, and *MCC* for all the methods. The *Sp* of the NetPhosBac was higher than the MPSite, but the *Sn* was very low (Table 3). The overall performance of the MPSite outperformed the NetPhosBac and others methods.

**Table 3:** Performance comparison of pS and pT site prediction on the independent dataset.

Method	*Sp*	*Sn*	*Ac*	*MCC*
	**Phospho-serine (pS)**			
MPSite	0.811	0.412	0.678	0.239
ANN	0.803	0.261	0.622	0.124
DT	0.801	0.291	0.631	0.157
NB	0.801	0.271	0.624	0.133
SVM	0.802	0.361	0.655	0.183
NetPhosBac	0.678	0.331	0.562	−0.006
	**Phospho-threonine (pT)**			
MPSite	0.818	0.616	0.751	0.432
ANN	0.806	0.465	0.692	0.292
DT	0.803	0.499	0.702	0.322
NB	0.801	0.446	0.683	0.283
SVM	0.805	0.565	0.725	0.372
NetPhosBac	0.883	0.101	0.622	0.011

In both of the pS and pT classifiers, the independent performances were lower than the original training dataset. This would be caused by the fact that the independent sets, definitely blind to the training set, are collected. Many published studies of PTM analysis have showed the same results^26,30,45^ that independent performances were lower than the training dataset.

While the MPSite achieved a promising performance, there is still room for enhanced prediction accuracy. In the proposed model we exclusively used the sequence information including a SSF feature to reduce calculation complexity, while recently the tertiary structure has been suggested as another key feature for PTM prediction^46^ and the exclusive use of sequence features may bias the prediction accuracy^47^. In the near future, we will utilize tertiary structural features to enhance prediction accuracy.

## Conclusions

An efficient computational approach was designed for identifying potential microbial pS and pT sites. We show that the MPSite is a promising method and provides an outstanding performance compared with existing methods. Analysis of the training and independent datasets demonstrated that the MPSite is useful for understanding the mechanisms of microbial phosphorylation sites. Finally, a user-friendly web application was developed and freely available for academic users.

## Supplementary information

Computational identification of microbial phosphorylation sites by the enhanced characteristics of sequence information
