# Supplementary material for: Computational identification of microbial phosphorylation sites by the enhanced characteristics of sequence information
Source: Sci Rep. 2019 Jun 4;9:8258. doi: 10.1038/s41598-019-44548-x (PMC6547684; doi:10.1038/s41598-019-44548-x)
Supplement: Supplementary file 1 — Computational identification of microbial phosphorylation sites by the enhanced characteristics of sequence information [file 41598_2019_44548_MOESM1_ESM.pdf]

Supplementary Information

Computational identification of microbial phosphorylation sites by the enhanced characteristics of sequence information

Md. Mehedi Hasan[1], Md. Mamunur Rashid[1], Mst. Shamima Khatun[1], and Hiroyuki Kurata[1,2*]

[1]Department of Bioscience and Bioinformatics, Kyushu Institute of Technology, 680-4 Kawazu, Iizuka, Fukuoka 820-8502, Japan.

[1,2]Biomedical Informatics R&D Center, Kyushu Institute of Technology, 680-4 Kawazu, Iizuka, Fukuoka 820-8502, Japan.

Table S1. 15 types of AIP properties.
They were used for pS and pT site prediction.

AIP properties	**Description**
BLAM930101	Alpha helix propensity
MAXF760101	Normalized frequency of alpha-helix
TSAJ990101	Volumes including the crystallographic
NAKH920108	AA composition of MEM of multi-spanning proteins
CEDJ970104	AA intracellular proteins
LIFS790101	Conformational preference for all beta-strands
NOZY710101	Transfer energy, organic solvent/water
HUTJ700103	Entropy of formation
NAKH900109	AA composition of membrane proteins
BIOV880101	Information value for accessibility
MIYS990104	Protein conformation structure
PUNT030101	Structural prediction of membrane-bound proteins
WOEC730101	Polar requirement
BASU050102	Eigenvector of contact matrices and hydrophobicity profiles
SUYM030101	Linker propensity

Table S2. Prediction performance for different positive versus negative samples in the training dataset.

The Ratio P/N	*Sp*	*Sn*	*Ac*	*MCC*	AUC
	Phospho-serine (pS)				
1:1	0.864	0.469	0.667	0.416	0.793
1:2	0.867	0.484	0.739	0.427	0.801
1:3	0.871	0.389	0.751	0.386	0.759
1:total	0.867	0.363	0.841	0.332	0.733
Phospho-threonine (pT)					
1:1	0.861	0.546	0.704	0.492	0.834
1:2	0.867	0.565	0.766	0.503	0.839
1:3	0.864	0.513	0.777	0.462	0.811
1:total	0.863	0.463	0.839	0.417	0.766

Table S3. Top 30 significant features selected by the WR scheme for pS site.
The p-values were measured by pair two-sample *t*-test between the positive and negative samples.

Selected Feature	WR features	*p*-value
1	v332 (AFC)	2.21E-05
2	v2882(AIP)	4.69E-21
3	v2441(AIP)	2.28E-22
4	v3764(PKA)	1.80E-23
5	v4205(PKA)	2.88E-19
6	v2431(AIP)	2.15E-17
7	v1541(AFC)	8.51E-19
8	v2872 (BE)	8.51E-19
9	v4562(BE)	9.61E-18
10	v4195(BE)	1.02E-17
11	v4604(BE)	3.02E-16
12	v4200(PKA)	7.18E-17
13	v3754(PKA)	3.57E-14
14	v3313(PKA)	1.84E-16
15	v4583(BE)	1.04E-16
16	v4625(BE)	7.62E-17
17	v4646(BE)	2.90E-16
18	v3759(PKA)	1.45E-14
19	v2877(PKA)	1.48E-12
20	v2318(AIP)	6.65E-13
21	v4667(BE)	1.60E-12
22	v2183(AIP)	3.37E-12
23	v222(AFC)	4.92E-14
24	v4688(BE)	1.44E-12
25	v2177(AIP)	1.15E-10
26	v232(AFC)	2.31E-12
27	v4233(PKA)	3.68E-10
28	v4709(BE)	1.75E-13
29	v4278(PKA)	1.07E-09
30	v3066(PKA)	1.12E-14

Table S4. Top 30 significant features selected by the WR scheme for pT site.
The *p*-values were measured by pair two-sample *t*-test between the positive and negative samples.

Number of Features	WR features	p-value
1	v244 (PKA)	2.95E-15
2	v2882 (AFC)	5.77E-14
3	v3323(AFC)	2.47E-12
4	v3764(AFC)	3.32E-10
5	v2431(AFC)	2.57E-13
6	v4641(BE)	2.57E-13
7	v4383(AIP)	2.32E-12
8	v3313(AFC)	6.63E-12
9	v4262(AIP)	1.83E-12
10	v2872(AFC)	4.96E-12
11	v322(PKA)	5.66E-12
12	v4704(BE)	1.54E-11
13	v3754(AFC)	4.35E-11
14	v2351(AFC)	4.00E-12
15	v4759(BE)	6.67E-09
16	v4200(AFC)	1.67E-08
17	v4305(AIP)	3.95E-08
18	v4625(BE)	6.54E-10
19	v4195(AIP)	2.34E-09
20	v4101(AFC)	9.34E-09
21	v437(PKA)	7.67E-09
22	v1522(PKA)	2.52E-09
23	v3760(AFC)	4.36E-08
24	v4248(AIP)	3.29E-12
25	v4062(AFC)	4.86E-09
26	v3319(AFC)	8.40E-07
27	v3318(AFC)	9.35E-07
28	v2752(AFC)	6.67E-10
29	v4646(BE)	8.58E-08
30	v4218(AIP)	1.01E-10
